# Kinetic insights into the temperature dependence of DNA strand cleavage and religation by topoisomerase III from the hyperthermophile *Sulfolobus solfataricus*

**DOI:** 10.1038/s41598-017-05837-5

**Published:** 2017-07-14

**Authors:** Junhua Zhang, Bailong Pan, Zhimeng Li, Xin Sheng Zhao, Li Huang

**Affiliations:** 1 State Key Laboratory of Microbial Resources, Institute of Microbiology, Chinese Academy of Sciences, Beijing, 100101 China; 2 Beijing National Laboratory for Molecular Sciences, State Key Laboratory for Structural Chemistry of Unstable and Stable Species, Department of Chemical Biology, College of Chemistry and Molecular Engineering, Peking University, Beijing, 100871 China; 3 Biodynamic Optical Imaging Center (BIOPIC), Peking University, Beijing, 100871 China; 4 College of Life Sciences, University of Chinese Academy of Sciences, Beijing, 100049 China

## Abstract

All cellular organisms encode type IA topoisomerases which catalyze DNA topological changes essential for DNA transactions. However, the kinetics of the reaction catalyzed by these enzymes remains poorly characterized. Here we measured the rapid kinetics of template binding, cleavage and religation by *Sso* topo III, a type IA topoisomerase from the hyperthermophilic archaeon *Sulfolobus solfataricus*, by using a novel FRET/PIFE-based method in a stopped-flow spectrometer. We show that *Sso* topo III bound the template rapidly, and the rate of binding was 2–3 orders of magnitudes higher than that of template cleavage at 25 °C. The rate of template cleavage was favored over that of template religation by the enzyme, and was more so at lower temperatures (25–55 °C). Significant template cleavage [(2.23 ± 0.11) × 10^−3^ s^−1^] was observed while little religation was detectable at 25 °C. This is consistent with the presence of a higher activation energy for template religation (41 ± 5 kcal·mol^−1^) than that for template cleavage (32 ± 1 kcal·mol^−1^). Our results provide a kinetic interpretation for the ability of *Sso* topo III to relax negatively supercoiled DNA only at higher temperature and offer clues to the adaptation of the reaction mechanisms of thermophilic enzymes to high temperature.

## Introduction

DNA topoisomerases are ubiquitous enzymes that catalyze topological changes in DNA essential for DNA transactions, such as DNA replication, transcription, DNA repair and recombination^1^. Type IA topoisomerases exist in all cellular organisms and include topoisomerases I and III as well as reverse gyrase^1^. These enzymes are capable of relaxing negative DNA supercoils, or introducing positive DNA supercoils in the case of reverse gyrase. Much of our current knowledge about the mechanistic aspects of type IA topoisomerases is derived from the studies of *Escherichia coli* topoisomerase I, a prototype of this subclass of topoisomerases^2, 3^. According to a well-accepted model, type IA topoisomerases relax DNA using an enzyme-bridged mechanism, which entails four steps in a catalytic cycle^4, 5^. The topoisomerase binds non-covalently to one strand in the single-stranded regions of DNA (step 1), and cleaves the strand non-randomly with the concomitant formation of a covalent linkage between the active-site tyrosyl of the enzyme and the scissile phosphate (step 2); the complementary strand passes through the transient opening on the cleaved strand (step 3), followed by the religation of the cleaved strand (step 4).

Topoisomerase III from the hyperthermophilic archaeon *Sulfolobus solfataricus* (*Sso* topo III) is a type IA topoisomerase. *Sso* topo III is most active in DNA relaxation at 75 °C, a temperature optimal for the growth of the organism^6^. Intriguingly, DNA cleavage and religation by *Sso* topo III differed significantly in temperature dependence. Significant DNA cleavage was observed at 25 °C, but relaxation of negatively supercoiled DNA became apparent only at temperatures above 50 °C. The enzyme was capable of efficiently rejoining the cleaved DNA strand only at higher temperatures.

Extensive studies have provided an in-depth view of the structural details of the reaction mechanism of type IA topoisomerases^7–11^. However, the kinetics of the individual steps in DNA relaxation by these enzymes is poorly understood. In particular, how the kinetics of the steps in *Sso* topo III-catalyzed reactions is affected by temperature remains to be determined. In this study, we developed a fluorescence-based method for monitoring the rapid kinetics of DNA cleavage and religation by a type IA topoisomerase in real time. Taking advantage of the thermophily of *Sso* topo III, we determined the rate constants of template cleavage and religation by the enzyme in a temperature range of 25~55 °C. Our results provide a kinetic interpretation for the ability of *Sso* topo III to relax negatively supercoiled DNA only at higher temperature.

## Results

### Temperature affects the Mg^2+^ dependence of DNA cleavage by *Sso* topo III

**Figure 1:**
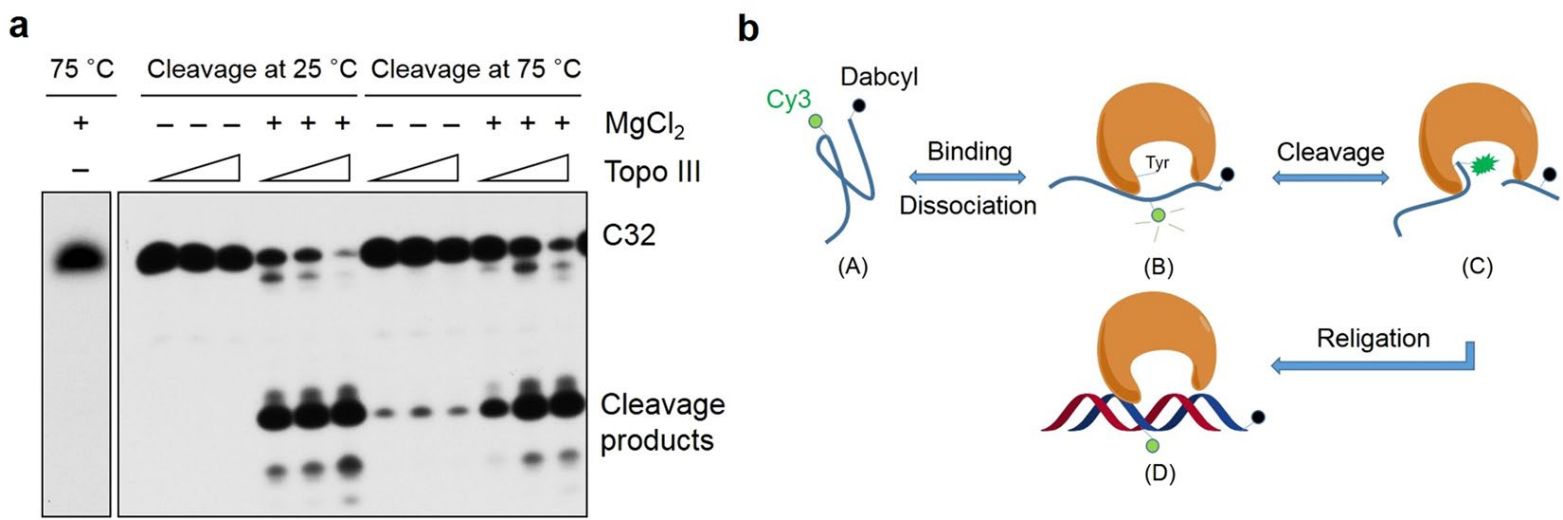
Activities of *Sso* topo III. (**a**) Effect of Mg^2+^ on C32 cleavage by *Sso* topo III at different temperatures. *Sso* topo III (32.6, 130 and 522 nM) was incubated for 15 min at 25 or 75 °C with ^32^P-labeled C32 (1.25 nM) in the standard cleavage assay mixture (0 or 2.5 mM MgCl_2_). Samples were resolved by electrophoresis in 18% urea-polyacrylamide gel. The gel was exposed to X-ray film and quantified by using ImageQuant software. The cleavage efficiencies were given in the text with three replicates evaluated. (**b**) A diagram depicting the detection of template binding, cleavage and religation by *Sso* topo III in the FRET/PIFE-based assay. (A) C25-Dabcyl-Cy3. (B) As *Sso* topo III binds to the template, the Cy3-to-Dabcyl distance increases, resulting in a Cy3 fluorescence increase due to the decrease of quenching efficiency by Dabcyl. (C) When the template is cleaved, the enzyme forms a covalent linkage with the Cy3-labeled 5′ end of the cleaved strand, generating the PIFE effect and exhibiting further increase of the Cy3 fluorescence intensity. (D) Annealing-promoted strand religation by *Sso* topo III occurs, accompanied by concomitant decrease of the fluorescence intensity of Cy3.

Type IA topoisomerases are known to depend on Mg^2+^ for the relaxation of negatively supercoiled DNA^12, 13^. To learn more about the effect of Mg^2+^ on individual steps in the process of DNA relaxation, we determined DNA cleavage by *Sso* topo III in the presence and the absence of MgCl_2_. An oligonucleotide template (C32), which contained a hot cleavage site (GCAAGGT↓CT), was used in the assay, and cleavage of ^32^P-labeled C32 by *Sso* topo III would produce a major product of 14 nt in size and a few minor bands^6^. As shown in Fig. 1a, wild-type *Sso* topo III (522 nM) was capable of significantly cleaving C32 in the absence of MgCl_2_ (~4% cleavage) although the cleavage activity was enhanced in the presence of MgCl_2_ (~50% cleavage) at 2.5 mM, the optimal concentration for the activity of the enzyme^14^, at 75 °C. By comparison, little cleavage was detected in the absence of MgCl_2_, whereas about 90% of the input DNA was cleaved by the enzyme in the presence of 2.5 mM MgCl_2_ at 25 °C. These results indicate that DNA cleavage by *Sso* topo III showed greater dependence on the presence of Mg^2+^ at temperatures significantly lower than that optimal for the growth of the organism.

### Development of a fluorescence-based method for the analysis of DNA binding, cleavage and religation by *Sso* topo III

**Figure 2:**
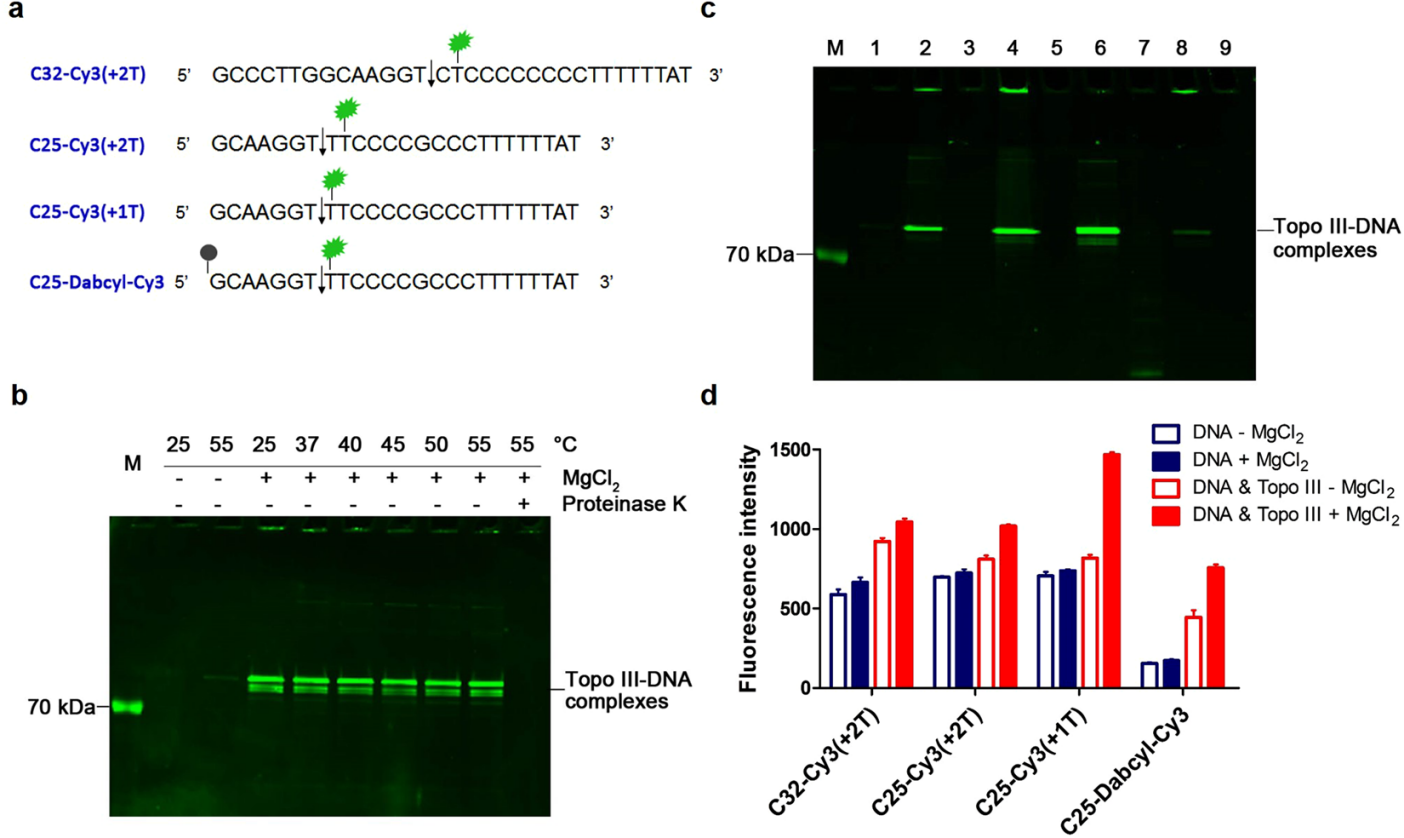
Test of fluorescence-labeled templates for the activity assays of *Sso* topo III. (**a**) The sequences of templates labeled with Cy3 (green) and Dabcyl (grey dot). The cleavage site for *Sso* topo III is shown by an arrow. (**b**) Fluorescence of the intermediates of the cleavage of C25-Dabcyl-Cy3 by *Sso* topo III. *Sso* topo III (320 nM) was incubated with C25-Dabcyl-Cy3 (8 nM) for 40 min at various temperatures. When indicated, the sample was treated with proteinase K (0.5 mg/ml) for 30 min at 50 °C. Samples were resolved by electrophoresis in 10% SDS-polyacrylamide gel. The gel was analyzed by a Typhoon scanner. The two bands migrating just behind the 70-kDa marker are the cleavage products. (**c**) Strand annealing-promoted cleaved strand religation by *Sso* topo III. *Sso* topo III (1 μM) was incubated with C25-Dabcyl-Cy3 (20 nM) for 40 min at 25 °C under the following conditions. Lane 1, no added MgCl_2_; lane 2, 1 mM MgCl_2_; lane 4, same as lane 2 except for the addition of 5 mM EDTA; lane 6, same as lane 4 except for the addition of NC25 at an NC25/C25-Dabcyl-Cy3 molar ratio of 3; lane 8, same as lane 6 except for the addition of 30 mM MgCl_2_; lanes 3, 5, 7 and 9, same as lanes 2, 4, 6 and 8 except that the samples were treated with proteinase K (0.5 mg/ml) as in (**b**). All samples were subjected to a process of cooling from 75 °C to 55 °C at 0.3 °C/s and subsequent incubation for 30 min at 55 °C. Samples were processed as in (**b**). (**d**) Fluorescence intensities of the tested templates under indicated conditions. Various dye-labeled templates (8 nM) were incubated with *Sso* topo III (320 nM) for 40 min at 25 °C in the presence or absence of 2.5 mM MgCl_2_. Samples were excited at 532 nm, and the intensity of Cy3 was analyzed by a Hitachi F-7000 fluorescence spectrophotometer. In the controls, free samples were treated in the same manner except for the omission of the enzyme. Data shown represent an average of three independent measurements.

In order to carry out rapid kinetic analysis of the reaction catalyzed by *Sso* topo III, we developed a novel assay based on protein induced fluorescence enhancement (PIFE) and fluorescence resonance energy transfer (FRET). For this assay, we designed the template C25-Dabcyl-Cy3, a C32-derived 25-nt oligonucleotide containing Dabcyl, a fluorescence quencher, at the 5′ end, and Cy3, a fluorescent dye attached to the base T at position +1 relative to the cleavage site (Figs 1b and 2a). The emission of Cy3 would report on its proximity to *Sso* topo III, and, when the enzyme gets close to Cy3, the rate at which the Cy3 isomerizes from the photo-active *trans* state to the photo-inactive *cis* state would decrease, resulting in an increase in quantum yield and thus an increase in the intensity of the fluorescence emission (the PIFE effect)^15–17^. Conceivably, when C25-Dabcyl-Cy3 is cleaved by *Sso* topo III, the Cy3 fluorophore located near the cleavage site would presumably be in close vicinity of the enzyme as the result of the formation of covalent linkage between the catalytic tyrosine (Y318) of the enzyme and the scissile phosphoryl of the template, and become brighter. On the other hand, binding of C25-Dabcyl-Cy3 by *Sso* topo III increases the fluorescence intensity of Cy3. We speculate that binding by the enzyme reduces the flexibility of the single-stranded template and, consequently, increases the distance between Cy3 and Dabcyl. As the distance between the Cy3 dye and the quencher Dabcyl becomes larger, the fluorescence of Cy3 gets stronger (the FRET effect). Therefore, the use of the fluorescence-labeled template C25-Dabcyl-Cy3 permits not only a PIFE-based analysis of the binding, but also a FRET-based analysis of the cleavage, of the template by *Sso* topo III. In this study, the rapid kinetics of template binding and cleavage by the enzyme was determined using this assay on a stopped-flow apparatus.

In our preliminary experiments, we incubated *Sso* topo III with C25-Dabcyl-Cy3 in the standard cleavage and religation reactions, and subjected the reaction products to SDS-PAGE. The template was readily cleaved, yielding covalent enzyme-DNA complexes (shown as bands migrating above the 70-kDa protein marker in Fig. 2b) as the reaction intermediate. When the protein covalently attached to the Cy3-labeled DNA was digested with proteinase K, the bands no longer existed (Fig. 2b). The cleaved C25-Dabcyl-Cy3 was religated, as revealed by a decrease in the fluorescence intensity of the enzyme-DNA band, when annealed to an excess of NC25 in a cooling process only in the presence of Mg^2+^, as expected^6^ (Fig. 2c, lane 8). In a separate control experiment, C11-Dabcyl-Cy3 (C11, a derivative of C25, contained the preferred site of cleavage for *Sso* topo III) was radiolabeled. The radiolabeled template was cleaved and religated as expected, as monitored by denaturing gel electrophoresis of the radiolabeled DNA, indicating that fluorescence labeling of the template with Cy3 and Dabcyl did not affect cleavage and religation of the template by the enzyme (Fig. S1). Therefore, we conclude that *Sso* topo III was able to bind, cleave and religate C25-Dabcyl-Cy3. We then tested if template C25-Dabcyl-Cy3 would permit the PIFE analysis of the cleavage and religation activities of *Sso* topo III as well as the FRET analysis of the binding ability of the enzyme. We first compared the changes in fluorescence in cleavage reactions, performed at 25 °C, using either C25-Cy3(+2 T) or C25-Cy3(+1 T) (i.e., C25 containing Cy3 attached to the base T at the second or the first position, respectively, downstream of the cleavage site) as the template in the presence and the absence of 2.5 mM MgCl_2_ (Fig. 2a). Mg^2+^ was required for significant DNA cleavage but not for DNA binding by *Sso* topo III at 25 °C (Figs 2b and S2). As shown in Fig. 2d, the fluorescence intensity of Cy3 in reactions containing C25-Cy3(+2 T) and *Sso* topo III in the presence of MgCl_2_ was slightly higher than that in the absence of MgCl_2_ (1.2 fold). However, a 2.0-fold increase in fluorescence intensity was recorded when C25-Cy3(+2 T) was replaced with C25-Cy3(+1 T) in the reaction. As a further control, Y318F, an active site mutant of *Sso* topo III^14^, instead of the wild type protein, was used with C25-Dabcyl-Cy3(+1 T) as the template in the assay. Y318F is unable to cleave DNA even in the presence of Mg^2+^ ^14^. The same fluorescence intensity was observed in the presence and the absence of Mg^2+^ (Fig. S3). These observations are consistent with the effect of PIFE. When C25-Dabcyl-Cy3(+1 T) was used as the template, the fluorescence intensity dropped dramatically in a protein-free control with or without Mg^2+^, compared to that in samples containing C25-Cy3(+1 T), due to the quenching effect of Dabcyl. Once *Sso* topo III was added to the reaction lacking Mg^2+^, the fluorescence signal of Cy3 was 3 fold as high as that in the absence of the enzyme, confirming that C25-Dabcyl-Cy3 can be used to determine template binding by the enzyme. Once cleavage by *Sso* topo III was initiated by the addition of MgCl_2_, the fluorescence intensity increased further, although not to the extent observed with C25-Cy3(+1 T) due to quenching by Dabcyl. Taken together, our results demonstrate that the fluorescence-based assay involving the use of C25-Dabcyl-Cy3 is well suited for the analysis of DNA binding, cleavage and religation by *Sso* topo III.

### Template binding by *Sso* topo III

**Figure 3:**
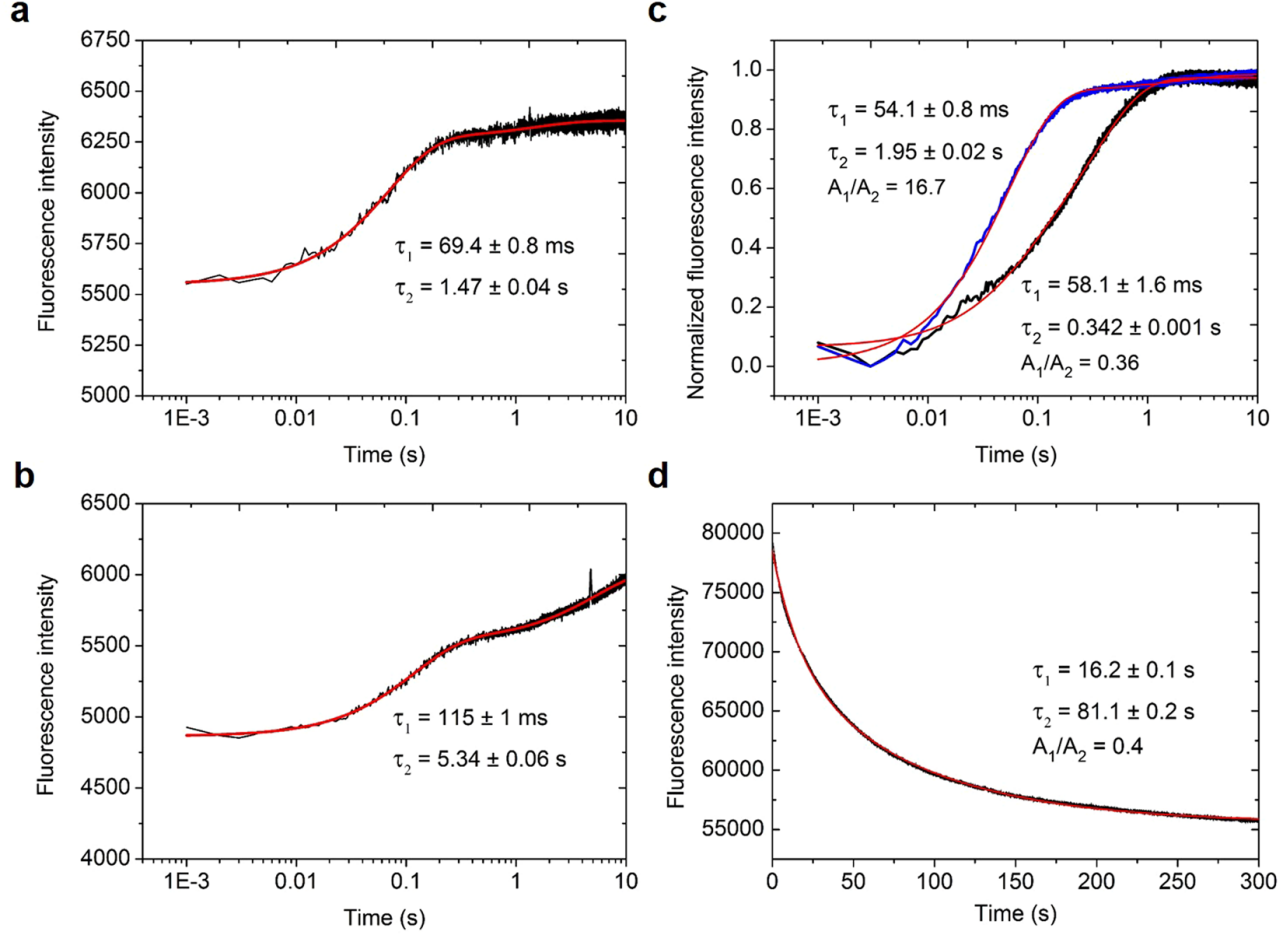
Kinetics of template binding and dissociation by Y318F at 25 °C. (**a** and **b**) Binding of C25-Dabcyl-Cy3 by Y318F. Y318F (320 nM) was mixed with C25-Dabcyl-Cy3 (8 nM) at 25 °C in the presence and absence of MgCl_2_ (2.5 mM), and the change in fluorescence was measured in a stopped-flow spectrometer. A fitted line, derived using a double-exponential function, is shown in red with time constants τ_1_ and τ_2_ indicated. (**c**) Comparison of binding of C11-Dabcyl-Cy3 and C11(dT)_14_-Dabcyl-Cy3 by Y318F. The graphs show the normalized time courses of binding of Y318F (20 nM) to the template (8 nM) in the presence of MgCl_2_ (2.5 mM). Two fitted lines, derived using a double-exponential function, are shown in red for C11 (black) and C11(dT)_14_ (blue), with respective time constants τ_1_ and τ_2_ indicated. (**d**) Dissociation of Y318F from C25-Dabcyl-Cy3. Y318F (50 nM) was first mixed with C25-Dabcyl-Cy3 (10 nM) at 25 °C in the presence of MgCl_2_ (2.5 mM). Unlabeled competitor C25 (450 nM) was added, and the change in fluorescence was measured in a stopped-flow spectrometer. A fitted line, derived using a double-exponential function, is shown in red with time constants τ_1_ and τ_2_ indicated. A_1_ and A_2_ are the amplitudes of the two variables in the double-exponential function. The A_1_/A_2_ ratio represents the relative weight of each of the two amplitudes. Each graph is derived from 5~6 independent experiments.

We then performed a stopped-flow analysis of DNA binding, cleavage and religation by *Sso* topo III using C25-Dabcyl-Cy3 as the template (Fig. S4). In the binding assay, Y318F was used to avoid the possible interference by the cleavage activity of the enzyme. As shown in Fig. 3a,b, the Cy3 intensity increased rapidly upon the mixing of Y318F with C25-Dabcyl-Cy3 at 25 °C in the presence and the absence of MgCl_2_. The change in fluorescence intensity in both cases fits a double-exponential function with a rapid increase in intensity in the first phase followed by a slower increase in intensity in the second phase. The rate of binding is described with time constant (τ), which refers to the time required to reach 1/e of the maximum Cy3 intensity. The time constants for the first phase (τ_1_), taken as a measure of the rate of template binding, were 69.4 ± 0.8 and 115 ± 1 ms, respectively, in the presence and absence of MgCl_2_, suggesting that template binding by Y318F was accelerated by MgCl_2_. The time constants for the second phase (τ_2_) were in the range of 1.4–5.4 s both in the presence and in the absence of MgCl_2_. The presence of the two phases in the process of binding by *Sso* topo III appears to indicate unknown conformational rearrangements that might take place in the formation of the protein-DNA complex^18^. It is possible that the first phase entails initial binding of the DNA by the enzyme and the second phase involves subsequent expansion of the contact between the enzyme and the DNA. Furthermore, the rate of template binding by Y318F appears to be similar to that by wild-type *Sso* topo III since the time constant for the first phase of the binding of the template by the wild-type protein in the absence of MgCl_2_ at 25 °C was also found to be in the millisecond range (τ_1_ = 178 ± 3 ms).

*Sso* topo III has a preferred cleavage sequence of G(A/T)CA(T)AG(T)G(A)X↓XX^14^. To determine how the sequences flanking the cleavage site might contribute to the binding of the target by the enzyme, we performed the binding assays on templates of two different sizes, i.e., C11-Dabcyl-Cy3 and C11(dT)_14_-Dabcyl-Cy3. Both fragments contained the preferred 9-nt cleavage sequence, and the latter template had a stretch of 14 T’s attached to the 3′ end of the former fragment (Fig. S5). We found that *Sso* topo III bound more poorly to a (dT)_32_ fragment (K_d_ = 42 nM) than to C32 (K_d_ = 16 nM) (Figs S5 and S6). Although the fitted second time constants (τ_2_) for the binding of the two templates differed significantly, the first time constants (τ_1_) for the binding of C11-Dabcyl-Cy3 and C11(dT)_14_-Dabcyl-Cy3 by Y318F at 25 °C in the presence of 2.5 mM MgCl_2_ were similar (58.1 ± 1.6 and 54.1 ± 0.8 ms, respectively), indicating that the low binding affinity sequence flanking the cleavage site barely affects the rate of template binding by the enzyme (Fig. 3c). The A_1_/A_2_ values for C11 and C11(dT)_14_ are 0.36 and 16.7, corresponding to the fluorescence intensities of (4.0 ± 0.1) × 10^3^ counts and (5.4 ± 0.2) × 10^3^ counts, respectively, after the first binding phase. The results suggest that the FRET efficiencies of the two DNA templates after the first binding phase are different, indicating that the conformations of the two templates during the binding are different. The different values of τ_2_ reflect the different rates of the conformational changes, further supporting the notion that the conformations of the two templates are different.

The rate of dissociation of the enzyme from the template was determined following the addition of unlabeled C25 to a preincubated mixture of Y318F and C25-Dabcyl-Cy3 in a large molar excess over the labeled template (Fig. 3d). The dissociation process was substantially slower than the binding process and occurred in two phases. The time constant for the early phase of the dissociation (τ_1_ = 16.2 ± 0.1 s) was smaller than that for the late phase (τ_2_ = 81.1 ± 0.2 s), implying a change in affinity of the protein for the template during the process of dissociation. The rate constant for template binding by the enzyme increased with an increasing temperature and became unmeasurable at temperatures higher than 25 °C with the instrument used in this study.

### Template cleavage is significantly slower than template binding by *Sso* topo III

**Figure 4:**
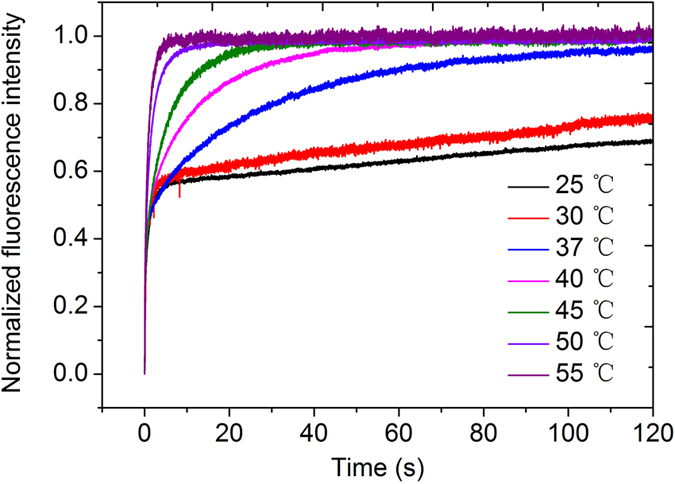
Cleavage of C25-Dabcyl-Cy3 by *Sso* topo III at various temperatures. Cleavage of C25-Dabcyl-Cy3 (8 nM) by *Sso* topo III (320 nM) in the presence of MgCl_2_ (2.5 mM) at 25, 30, 37, 40, 45, 50 or 55 °C was performed, and the change in fluorescence was measured in a stopped-flow spectrometer. The data were fitted to double-exponential function. Each curve is derived from 5~6 independent measurements.

To examine the rate of cleavage by *Sso* topo III, we rapidly mixed the enzyme with C25-Dabcyl-Cy3 at various temperatures in the presence of 2.5 mM MgCl_2_ and followed the time course of the change in Cy3 fluorescence (Fig. 4). The fluorescence intensity changed in two distinct phases: a sharp increase followed by a slow increase. The initial increase appeared to have resulted from the binding of the enzyme to the template since the time constant of change (132 ± 20 ms) was similar to the time constant of binding (69.4 ± 0.8 ms) by the enzyme at 25 °C. The slower increase in the second phase was due to template cleavage, which involved the breakage of a phosphodiester bond, by the enzyme. Once the template was cleaved, the enzyme formed a covalent linkage with the Cy3-labeled 5′ end of the cleaved strand, producing the PIFE effect. By fitting the double-exponential function, the time constant of strand cleavage was calculated to be 448 ± 22 s. A similar rate of cleavage was found when the process of the cleavage reaction was monitored by SDS-PAGE analysis of the reaction intermediate (Fig. S7). Therefore, the observed rate constant of cleavage (*k*_clv,obs_) was (2.23 ± 0.11) × 10^−3^ s^−1^ at 25 °C. We also measured rates of the cleavage of C25-Dabcyl-Cy3 by *Sso* topo III at higher temperatures. The time constants were 201 ± 5, 32.7 ± 1.6, 15.6 ± 1.0, 7.35 ± 0.44, 3.19 ± 0.30 and 1.55 ± 0.10 s at 30, 37, 40, 45, 50 and 55 °C, respectively (Fig. 4, Table 1). Consequently, the observed rate constants for DNA cleavage (*k*_clv,obs_) were (4.98 ± 0.12) × 10^−3^, 0.0306 ± 0.0015, 0.0641 ± 0.0041, 0.136 ± 0.008, 0.313 ± 0.029, 0.645 ± 0.042 s^−1^ at 30, 37, 40, 45, 50 and 55 °C, respectively. Therefore, template cleavage by *Sso* topo III accelerated as temperature increased within the tested range. However, strand cleavage was slower than strand binding by the enzyme.

**Table 1:** Kinetic parameters for strand cleavage and religation by *Sso* topo III at different temperatures.

Temp (°C)	*K*_L_	*k*_clv,obs_ (s^−1^)	*k*_reli_ (s^−1^)	*k*_clv_ (s^−1^)
25	—	(2.23 ± 0.11) × 10^−3^	—	
30	—	(4.98 ± 0.12) × 10^−3^	—	
37	0.0512 ± 0.0048	0.0306 ± 0.0015	(1.49 ± 0.08) × 10^−3^	0.0291 ± 0.0023
40	0.0843 ± 0.0072	0.0641 ± 0.0041	(4.98 ± 0.22) × 10^−3^	0.0591 ± 0.0043
45	0.0619 ± 0.0045	0.136 ± 0.008	(7.94 ± 0.12) × 10^−3^	0.128 ± 0.009
50	0.0644 ± 0.0001	0.313 ± 0.029	0.0189 ± 0.0070	0.294 ± 0.030
55	0.156 ± 0.001	0.645 ± 0.042	0.0870 ± 0.0080	0.558 ± 0.050

Data shown represent an average of 5~6 independent measurements.

### Template religation by *Sso* topo III is favored at higher temperature

**Figure 5:**
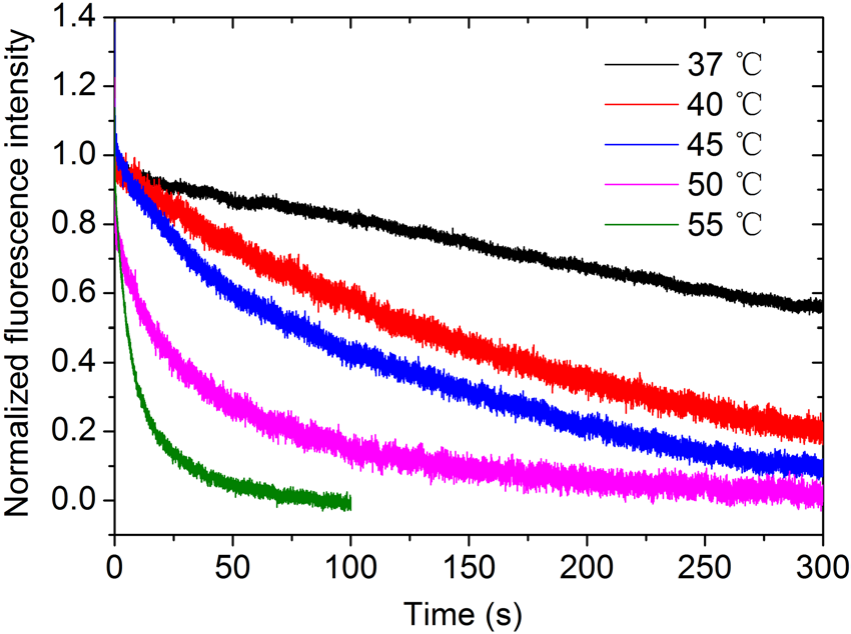
Annealing-promoted religation of the cleaved C25-Dabcyl-Cy3 DNA by *Sso* topo III at various temperatures. *Sso* topo III (2 μM) was incubated with C25-Dabcyl-Cy3 (40 nM) for 40 min at 25 °C in the presence of MgCl_2_ (1 mM). EDTA (5 mM) was added to chelate Mg^2+^. The cleavage intermediates were allowed to cool down from 75 °C to indicated temperatures at 0.3 °C/s in the presence of EDTA (5 mM) and the complementary strand NC25 at an NC25/C25-Dabcyl-Cy3 molar ratio of 3. Religation of the cleaved strand was initiated by the addition of MgCl_2_ (30 mM) at 37, 40, 45, 50 or 55 °C, and the change in fluorescence was measured in a stopped-flow spectrometer. The data were fitted to first-order exponential function. Each curve is derived from 5~6 independent measurements.

We showed previously that template DNA cleaved by *Sso* topo III was religated once annealed to a complementary non-cleaved strand^6^. Taking advantage of this observation, we first cleaved C25-Dabcyl-Cy3 with *Sso* topo III at 25 °C in the presence of 1 mM MgCl_2_, and then added EDTA to chelate Mg^2+^. An excess of the complementary NC25 was subsequently added, and the temperature of the mixture was raised to 75 °C and cooled down slowly from 75 °C to a specified temperature to allow strand annealing to occur. Religation of the cleaved intermediate was initiated by the addition of MgCl_2_ in the stopped-flow spectrometer, and the change of the Cy3 fluorescence intensity was recorded. Since strand religation occurred concomitantly with the breakage of the covalent linkage between the enzyme and the Cy3-labeled nucleotide, a decrease in the fluorescence intensity would be expected. Indeed, we found that the fluorescence emission from Cy3 decreased dramatically upon the addition of MgCl_2_ (Fig. 5). The time-course curves were fitted by the first-order exponential function, and the time constants for template religation were calculated to be 671 ± 36, 201 ± 9, 126 ± 2, 52.9 ± 19.6 and 11.5 ± 1.1 s at 37, 40, 45, 50 and 55 °C, respectively. Correspondingly, the rate constants for religation (*k*_reli_) were (1.49 ± 0.08) × 10^−3^, (4.98 ± 0.22) × 10^−3^, (7.94 ± 0.12) × 10^−3^, 0.0189 ± 0.0070 and 0.0870 ± 0.0080 s^−1^ at 37, 40, 45, 50 and 55 °C, respectively (Table 1). The rates of religation were drastically slower than the rates of cleavage by the enzyme at 25 and 30 °C, and were too slow to be detected by the stopped-flow method.

DNA cleavage and religation by *Sso* topo III are in equilibrium in the absence of the non-cleaved strand^6^. The equilibrium constant between the cleavage and religation (*K*_L_) is defined as:

1 $${K}_{{\rm{L}}}=\,\frac{{k}_{{\rm{r}}{\rm{e}}{\rm{l}}{\rm{i}}}}{{k}_{{\rm{c}}{\rm{l}}{\rm{v}}}}$$

where *k*_clv_ and *k*_reli_ represent the rate constants of cleavage and religation reactions, respectively. Assuming that the addition of the non-cleaved strand did not alter the rate of religation and only blocked the cleavage reaction, the true rate constant of cleavage is derived as follows.

2 $${{k}}_{{\rm{clv}}}=\,{{k}}_{{\rm{clv,obs}}}-\,{{k}}_{{\rm{reli}}}$$

The rate constants for religation (*k*_reli_), the true rate constants for cleavage (*k*_clv_) calculated by equation (2) and the equilibrium constants of the reactions (*K*_L_) at different temperatures are summarized in Table 1. *K*_L_ was very small at 37 °C (0.0512 ± 0.0048), indicating that cleavage reaction was overwhelmingly favored at this temperature. As the equilibrium constant became larger with an increasing temperature up to 55 °C, a greater proportion of the template DNA existed in an uncleaved or religated form at equilibrium. *K*_L_ (0.156 ± 0.001) at 55 °C was three times as high as that at 37 °C.

**Figure 6:**
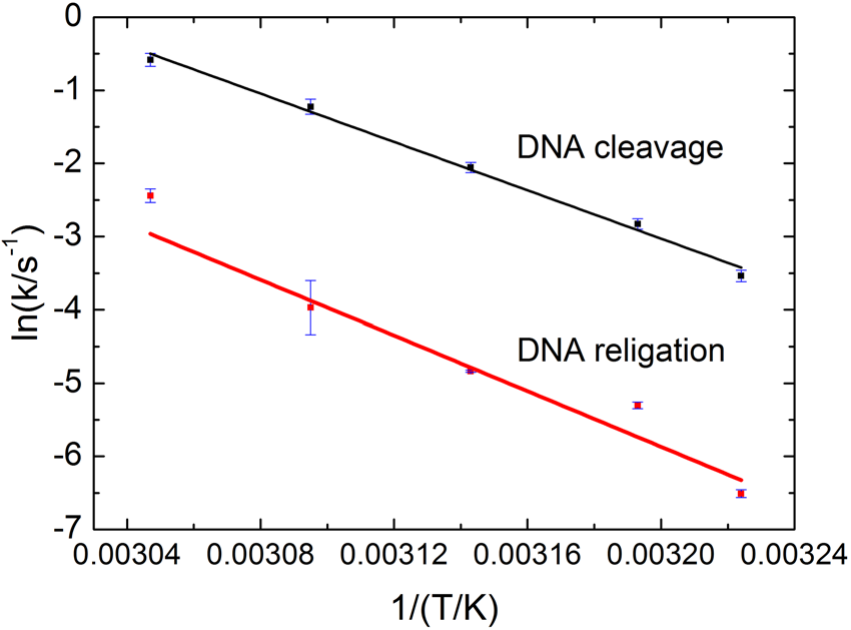
Apparent activation energies for cleavage and religation reactions catalyzed by *Sso* topo III. Each data point shown represents an average of 5~6 independent measurements.

By plotting rate constants against temperatures using the Arrhenius equation

3 $${\rm{l}}{\rm{n}}\,k=-{E}_{{\rm{a}}}/RT+{\rm{l}}{\rm{n}}A$$

the apparent activation energies (*E*_a_) for DNA cleavage and religation by *Sso* topo III were determined to be 32 ± 1 and 41 ± 5 kcal·mol^−1^, respectively (Fig. 6). Since the *E*_a_ of the religation reaction was larger than that of the cleavage reaction, religation was enhanced at higher temperatures. Although it was not possible to measure the kinetic constants of the reaction at temperatures higher than 55 °C using the stopped-flow spectrometer employed in this study, we were able to extrapolate a *k*_clv_ value of 10.9 s^−1^ and *k*_reli_ value of 2.62 s^−1^ for the reaction at 75 °C, the optimal growth temperature for the organism, according to the Arrhenius equation. Clearly, both cleavage and religation reactions would proceed efficiently at this temperature.

## Discussion

Although type IA topoisomerases have been extensively studied, very little is known about the kinetics of individual steps in the reaction cycle catalyzed by these enzymes. In this report, we developed a FRET- and PIFE-based topoisomerase assay, which permitted the determination of the rapid kinetics of template binding, cleavage and religation by *Sso* topo III, in conjunction with the use of a stopped-flow spectrometer. In addition, the thermophily of the protein made it possible for us to study the temperature dependence of the cleavage and religation kinetics of the topoisomerase. We show that template binding by *Sso* topo III was about three orders of magnitude faster than template cleavage by the enzyme at 25 °C. Although the rate of DNA cleavage by *Sso* topo III increased with an increasing temperature, template binding by the enzyme accelerated with temperature as well. And the rate of template binding was substantially higher than that of template cleavage for the enzyme within the range of temperatures tested. This observation contrasts with the finding that the integrase from SSV1, a virus infecting *Sulfolobus shibatae*, bound the template more slowly than cleaving it in an only kinetic study on a thermophilic tyrosine recombinase^19^. The discrepancy may be attributed to the difference in target sequence specificity of the two enzymes, with *Sso* topo III showing less sequence preference than SSV1 integrase in template binding.

The rates of both template cleavage and template religation by *Sso* topo III depended strongly on temperature in a tested range from 25 to 55 °C. No kinetic measurements have been reported on type IA topoisomerases yet. However, several type IB topoisomerases, which differ from type IA enzymes in forming a cleavage intermediate in which the cleaved strand is covalently linked to the active site of the enzyme, have been subjected to kinetic analyses. The rate constants of template cleavage and religation for human topoisomerase I at 37 °C are 69 × 10^−4^ and 9.5 × 10^−4^ s^−1^, respectively^20^, whereas the two parameters for Vaccinia topoisomerase I at 37 °C are 0.04 and 0.12 s^−1^, respectively^21^. By comparison, the *k*_clv_ and the *k*_reli_ of *Sso* topo III at 37 °C were 0.0291 and 1.49 × 10^−3^ s^−1^, respectively. It appears that the *Sulfolobus* enzyme and the two type IB enzymes are on the same order of magnitude in catalytic efficiency. It would be of interest to measure the kinetics of mesophilic type IA topoisomerases to determine if a thermophilic topoisomerase, such as *Sso* topo III, might have gained thermal stability without having to lose the catalytic efficiency, as implied in the above comparative analyses. The *k*_clv_ and the *k*_reli_ of *Sso* topo III at 75 °C, the temperature close to that optimal for the growth of the organism, are estimated to be 10.9 and 2.62 s^−1^, respectively, by extrapolation from the data obtained at lower temperatures. Therefore, the *Sulfolobus* topoisomerase is far more catalytically active than the two type IB enzyme, when they are compared at the optimal temperatures of the source organisms.

The equilibrium between cleavage and religation of the template by *Sso* topo III was tilted toward the former, and was more so at a lower temperature. The equilibrium constant of the reactions (*K*_L_) increased from 0.0512 ± 0.0048 at 37 °C to 0.156 ± 0.001 at 55 °C, with an extrapolated value of 0.240 at 75 °C. Because of the difference in activation energy between the cleavage reaction (32 ± 1 kcal·mol^−1^) and the religation reaction (41 ± 5 kcal·mol^−1^), religation became more favored at higher temperature. This provides an explanation for the finding that *Sso* topo III was capable of significant DNA cleavage at 25 °C but relaxed negatively supercoiled DNA only at higher temperature^6^. However, it should be noted that single-stranded template was used as the template for the cleavage and religation assay in this study. The presence of the complementary strand in double-stranded DNA will pull the reaction equilibrium in the direction of religation by reducing the accessibility of the enzyme to its single-stranded target sequence.

## Materials and Methods

### Proteins

The expression vector for *Sso* topo III (pET30a-topo III) was described previously^6^. The active site mutant of *Sso* topo III (Y318F) was constructed by site-directed mutagenesis using the QuickChange mutagenesis kit (TransGen BioTech, China) with pET30a-topo III as the template (see Table S1 for primers). Both wild-type *Sso* topo III and Y318F were overproduced as a C-terminally His6-tagged protein in *E. coli* strain Rosetta 2(DE3)pLysS. Wild-type and mutant *Sso* topo III proteins are overproduced and purified as described previously^6^.

### Oligonucleotide templates

Oligonucleotide C32 (5′-GCCCTTGGCAAGGTCTCCCCCCCCTTTTTTAT-3′) (the major cleavage sequence is underlined) was employed as the cleaved strand for *Sso* topo III. The sequences of additional templates derived from C32, e.g., C25, are shown in figure legends. NC25, an oligonucleotide complementary to C25 in sequence, was used as the non-cleaved strand. Oligonucleotides were labeled at the 5′ end with [γ-^32^P]ATP using T4 polynucleotide kinase. Templates for kinetic measurements were synthesized with a quencher Dabcyl attached to the base at the 5′ end and/or a fluorescent dye Cy3 at the base T on the 3′ side of the major cleavage site at Sangon BioTech (Shanghai, China) (Table S1).

### DNA cleavage and religation reactions

DNA cleavage and religation by *Sso* Topo III were performed as described previously^6^. Briefly, the cleavage reaction was carried out by incubating the enzyme with a radiolabeled oligonucleotide or fluorescence-labeled template in 50 mM Tris-HCl, pH 8.8, 0.1 mM EDTA, pH 8.0, 2.5 mM MgCl_2_, 90 mM NaCl and 30 μg/ml BSA at an indicated temperature. Religation of the cleaved template by the enzyme was effected by annealing unlabeled non-cleaved strand to the cleaved strand in a Veriti thermal cycler (Applied Biosystems, USA), or by the addition of 0.6 M NaCl. Reactions were terminated with 0.5% SDS. For reactions using a radiolabeled template, samples were subjected to electrophoresis in 18% polyacrylamide (19:1) gel containing 8 M urea in 0.5 × TBE buffer. The gel was exposed to X-ray film. For reactions using a fluorescence-labeled template, samples were subjected to electrophoresis in 10% SDS polyacrylamide gel in 1 × Tris-glycine buffer. The gel was imaged on a Typhoon scanner (GE Healthcare).

### Fluorescence measurements

*Sso* topo III was incubated for 40 min at 25 °C with a Cy3-labeled oligonucleotide template in 50 mM Tris-HCl, pH 8.8, 0.1 mM EDTA, pH 8.0, 90 mM NaCl and 30 μg/ml BSA in the presence or absence of 2.5 mM MgCl_2_. The fluorescence spectrum of Cy3 excited at 532 nm was immediately taken between 550 and 750 nm on a Hitachi fluorescence spectrophotometer (F-7000).

### Kinetic measurements

Kinetic experiments were conducted on a homebuilt stopped-flow apparatus based on the SFM-300 stopped-flow spectrometer module (Bio-logic, France) equipped with a 532 nm CW Ya-Ge laser (SUW Tech., China) as the light source, a confocal optical path and an APD detector. The stopped-flow unit and the observation cell with a 1.5 mm path length were thermostated by circulating water from a temperature-controlled bath. The dead time of the instrument was estimated to be 2.4 ms. Each component was detected by a photon-counting avalanche photodiode (APD) (SPCM-AQRH-14, Perkin-Elmer Optoelectronics) after passing through a filter (Semrock 595/50). The kinetics of DNA binding by *Sso* topo III was determined by rapidly mixing equal volumes (50 μl) of Cy3-Dabcyl-labeled C25 DNA (8 nM) and Y318F (320 nM) in buffer A 50 mM Tris-HCl, pH 8.8, 90 mM NaCl, 0.1 mM EDTA, pH 8.0, 2.5 mM MgCl_2_ and 0.01% Tween 20 (Sigma, Germany) on the stopped-flow apparatus at an indicated temperature. The rate constant of the dissociation of *Sso* topo III from the template was determined by incubating Y318F (500 nM) for 40 min with Cy3-Dabcyl-labeled C25 DNA (100 nM) in buffer A, and subsequently adding unlabeled C25 to an unlabeled C25/labeled 25 ratio of 45. To determine DNA cleavage kinetics, C25-Dabcyl-Cy3, *Sso* topo III and MgCl_2_ were rapidly mixed at a volume ratio of 2:2:1 to final concentrations of 8 nM, 320 nM and 2.5 mM, respectively, in buffer A. To measure DNA religation kinetics, *Sso* topo III (2 μM) was first incubated with 40 nM Dabcyl-Cy3-C25 for 40 min at 25 °C in buffer B 50 mM Tris-HCl, pH 8.8, 90 mM NaCl, 0.1 mM EDTA, pH 8.0, 1 mM MgCl_2_ and 0.01% Tween 20 (Sigma, Germany). EDTA (5 mM) was added to chelate Mg^2+^. A 3-fold molar excess of the complementary strand NC25 was then added to allow annealing to take place, as described previously^6^. Equal volumes of the reaction mixture and MgCl_2_ (30 mM) were rapidly mixed to initiate strand religation. For florescence resonance energy transfer (FRET) measurements in these reactions, the Cy3 dye was excited at 532 nm and the fluorescence emission was detected using a 595/50 filter (Semrock, USA). The kinetic traces represented an average of five to six individual scans and were fitted to exponential functions.

## Electronic supplementary material

Kinetic insights into the temperature dependence of DNA strand cleavage and religation by topoisomerase III from the hyperthermophile Sulfolobus solfataricus
