# Supplementary material for: Kinetic insights into the temperature dependence of DNA strand cleavage and religation by topoisomerase III from the hyperthermophile Sulfolobus solfataricus
Source: Sci Rep. 2017 Jul 14;7:5494. doi: 10.1038/s41598-017-05837-5 (PMC5511271; doi:10.1038/s41598-017-05837-5)
Supplement: Supplementary file 1 — Kinetic insights into the temperature dependence of DNA strand cleavage and religation by topoisomerase III from the hyperthermophile Sulfolobus solfataricus [file 41598_2017_5837_MOESM1_ESM.pdf]

Supplementary information for

Kinetic insights into the temperature dependence of DNA strand cleavage and religation by topoisomerase III from the hyperthermophile *Sulfolobus solfataricus*

Junhua Zhang[1,+], Bailong Pan[2,3,+], Zhimeng Li[1], Xin Sheng Zhao[2,3,*], and Li Huang[1,4,*]

[1]State Key Laboratory of Microbial Resources, Institute of Microbiology, Chinese Academy of Sciences, Beijing, 100101, China

[2]Beijing National Laboratory for Molecular Sciences, State Key Laboratory for Structural Chemistry of Unstable and Stable Species, Department of Chemical Biology, College of Chemistry and Molecular Engineering, Peking University, Beijing, 100871, China

[3]Biodynamic Optical Imaging Center (BIOPIC), Peking University, Beijing, 100871, China

[4]College of Life Sciences, University of Chinese Academy of Sciences, Beijing, 100049, China

[*]Corresponding authors

Fax: +86 10 62751708, Tel: +86 10 62751727, e-mail: zhaoxs@pku.edu.cn

Fax: +86 10 64807429; Tel: +86 10 64807430, e-mail: huangl@sun.im.ac.cn

[+]These authors contributed equally to this work.

Table S1. Oligonucleotides and primers used in this study.

Construct	Name	Sequence (5’ - 3’)
**Template**	C32	GCCCTTGGCAAGGTCTCCCCCCCCTTTTTTAT
	C25	GCAAGGTTTCCCCGCCCTTTTTTAT
	NC25	ATAAAAAAGGGCGGGGAAACCTTGC
	C11	GCAAGGTTTCC
	$(dT)_{32}$	TTTTTTTTTTTTTTTTTTTTTTTTTTTTTTTT
	$C11(dT)_{14}$	GCAAGGTTTCCTTTTTTTTTTTTTT
***Sso* topo III**	Topo III-F	GGAATTCCATATGAATTTATGTAATGTAAACAAC
	Topo III-R	CCGCTCGAGTTCACTGCTTAGCATATAAGTTAC
**Y318F**	Y318F-F	ACGGTCTAATAAGTTTCCCAAGAACT
	Y318F-R	AAACTTATTAGACCGTCCAAATAAAG

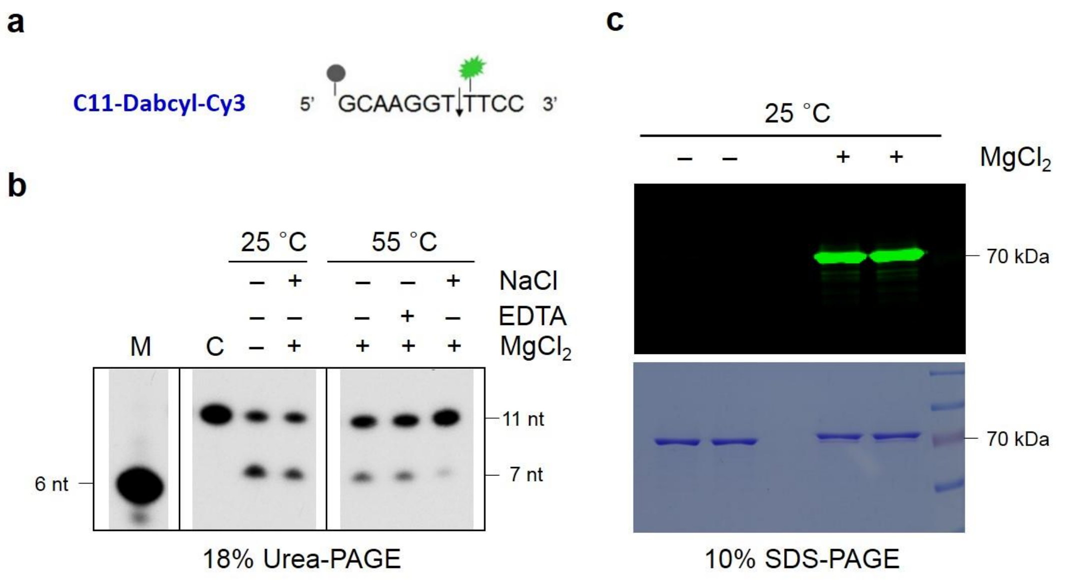

Figure S1. Cleavage of ^{32}P-labeled C11 and C11-Dabcyl-Cy3 by *Sso* topo III. (a) C11-Dabcyl-Cy3. Oligonucleotide C11, containing Cy3 and Dabcyl. The fluorophore Cy3 (green) and the fluorescence quencher Dabcyl (grey dot) are indicated. The cleavage site for *Sso* topo III is shown by an arrow. (b) Effect of Mg^{2+} on C11 cleavage and religation by *Sso* topo III at different temperatures. *Sso* topo III (65 nM) was incubated for 15 min at 25 or 55 ℃ with ^{32}P-labeled C11 (1.25 nM) in the standard cleavage assay mixture in the presence or absence of 2.5 mM $MgCl_2$. NaCl (0.6 M) was added to initiate religation of the cleaved template in some samples, while EDTA (5 mM) was added to chelate Mg^{2+} in other samples, as indicated. C, ^{32}P-labeled C11; M, a ^{32}P-labeled 6-nt oligonucleotide. Samples were resolved by electrophoresis in 18% urea-polyacrylamide gel. (c) Fluorescence of the intermediate of cleavage of C11-Dabcyl-Cy3 by *Sso* topo III. *Sso* topo III (320 nM) was incubated with C11-Dabcyl-Cy3 (8 nM) for 40 min at 25 ℃. Samples were resolved by electrophoresis in 10% SDS-polyacrylamide gel. The gel was analyzed by a Typhoon scanner (upper panel) and Coomassie brilliant blue staining (lower panel).

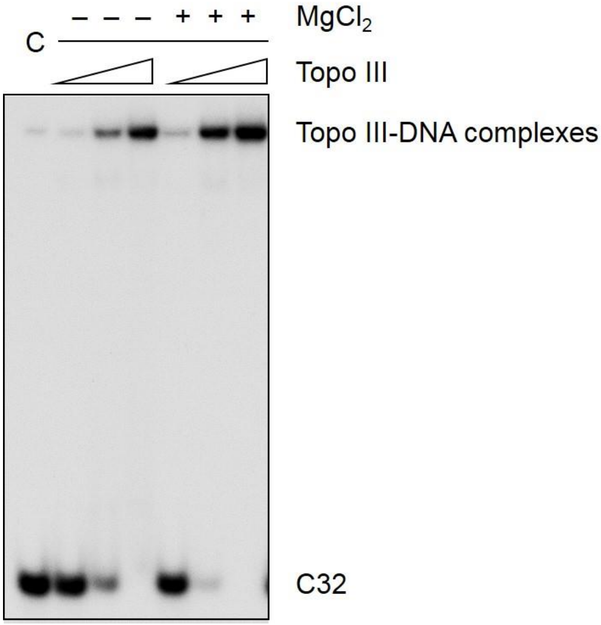

Figure S2. Binding of C32 by *Sso* topo III. *Sso* topo III was serially diluted (65.3, 16.3 and 4.1 nM) and mixed with ^{32}P-labeled C32 (1.25 nM) in the standard assay mixture in the presence or absence of 2.5 mM $MgCl_2$. Incubation was at 25 ℃ for 5 min. Samples were analyzed by electrophoresis in 8% polyacrylamide gel. The gel was exposed to X-ray film. Lane C, no enzyme.

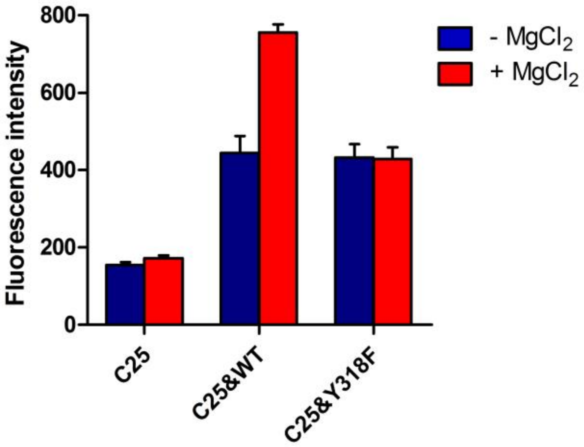

Figure S3. Detection of the template cleavage of *Sso* topo III by Cy3 fluorescence measurement. *Sso* topo III (320 nM, WT) or Y318F (320 nM) was incubated with C25-Dabcyl-Cy3 (8 nM) for 40 min at 25 ℃ in the presence (red) or the absence (blue) of 2.5 mM $MgCl_2$. The fluorescence of Cy3 was measured. As a control, samples without the enzyme are shown. Data shown represent an average of three independent measurements.

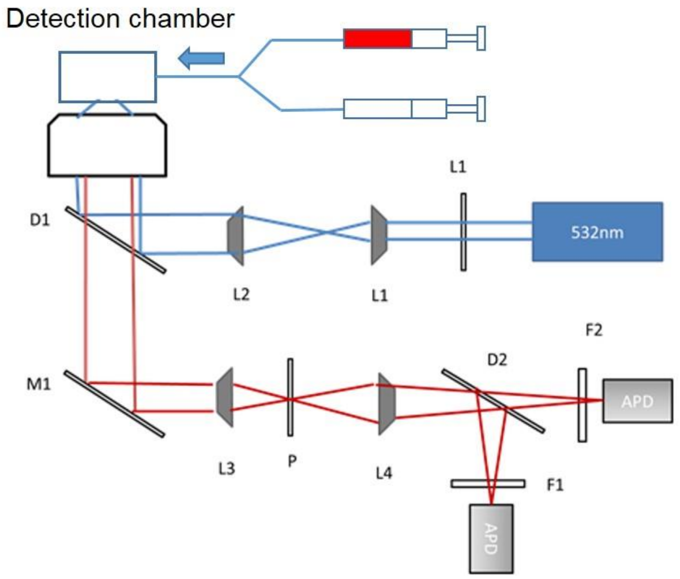

Figure S4. Diagram of the stopped-flow instrument. The instrument was modified based on a stopped-flow mixing system (SFM-300, BioLogic). As shown in this diagram, a small volume of each of the two solutions, one of which contains the Cy3-labeled template, is rapidly applied by a syringe and passed through a mixer to initiate the reaction. This mixture then goes into a detection chamber, and the flow is stopped. The fluorescence of the mixture is detected in the detection chamber. The time interval between the mixing of the solutions and the beginning of signal detection, referred to as the ‘dead time’, is about 2.4 ms. The fluorescence intensity was determined by using the detecting light path of a confocal microscope (L, lens; F, filter; D, dichroic mirror, M, plane mirror; P, pinhole; APD, avalanche photodiode).

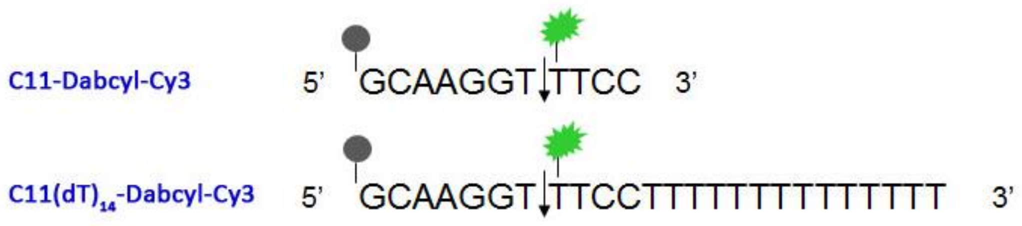

Figure S5. Fluorescence-labeled templates used in the template binding assay. C11 and C11(dT)$_{14}$ labeled with Cy3 (green) and Dabcyl (grey dot) are shown. The cleavage site for *Sso* topo III is indicated by an arrow.

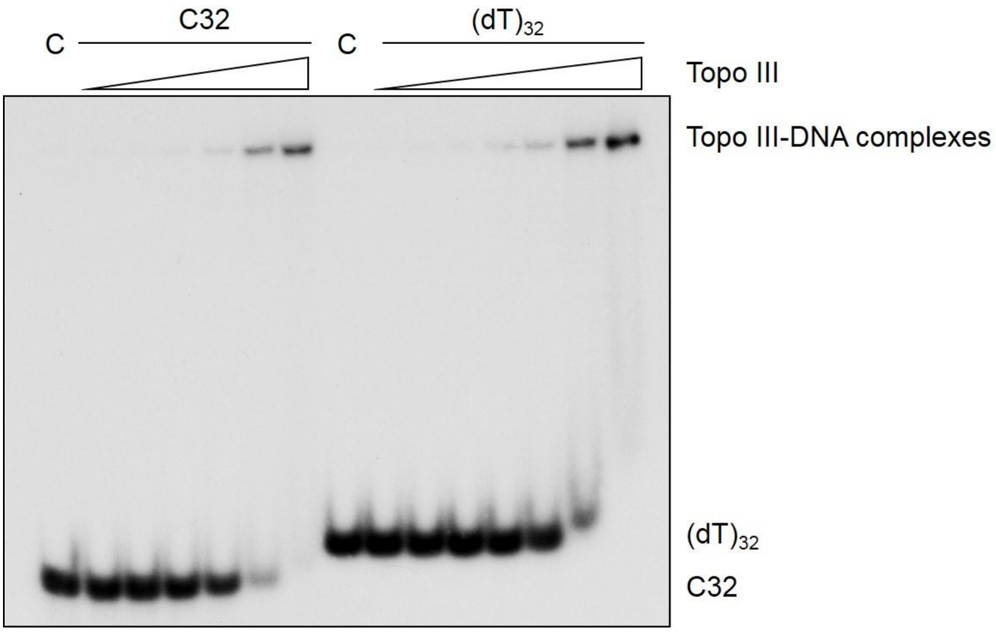

Figure S6. Binding of C32 and $(dT)_{32}$ by *Sso* topo III. *Sso* topo III was serially diluted (65.3, 32.6, 16.3, 8.2, 4.1 and 2.0 nM) and mixed with ^{32}P-labeled C32 (1.25 nM) in the standard assay mixture in the presence of 2.5 mM $MgCl_2$. *Sso* topo III was serially diluted (131, 65.3, 32.6, 16.3, 8.2, 4.1 and 2.0 nM) and mixed with ^{32}P-labeled C32 (1.25 nM) in the standard assay mixture in the presence of 2.5 mM $MgCl_2$. Incubation was at 25 ℃ for 5 min. Samples were analyzed by electrophoresis in 8% native polyacrylamide gel. The gel was exposed to X-ray film. Lane C, no enzyme.

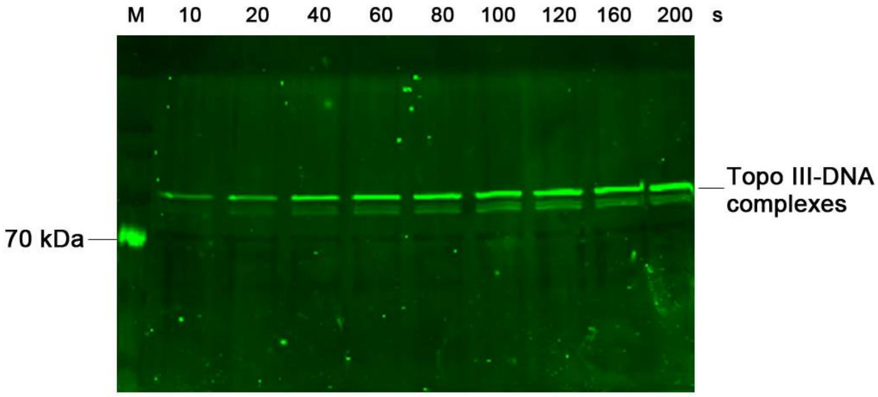

Figure S7. Time course of template cleavage by *Sso* topo III. *Sso* topo III (320 nM) was mixed with C25-Dabcyl-Cy3 (8 nM) and $MgCl_2$ (2.5 mM) at 25 ^oC. Aliquots were taken, and the reaction was terminated by the addition of EDTA (250 mM) at indicated time points (10, 20, 40, 60, 80, 100, 120, 160 and 200 s). Samples were resolved by electrophoresis in 10% SDS-polyacrylamide gel. The gel was analyzed by a Typhoon scanner.
